# Cross-level moderating effects of teacher experience, teacher evaluation, and teacher gender on student BMI status and peer relationships among Chinese middle school students

**DOI:** 10.3389/fpsyg.2026.1709999

**Published:** 2026-02-10

**Authors:** Keyi Liu, Chenglong Miao

**Affiliations:** 1 Department of Physical Education, Korea University, Seoul, Republic of Korea; 2 Department of Leisure Sports, Kangwon National University, Samcheok, Republic of Korea

**Keywords:** Chinese middle school students, HLM, teacher experience, teacher evaluation, teacher gender, student BMI status, peer relationships

## Abstract

**Objective:**

This study employed Hierarchical Linear Modeling to examine the moderating roles of teacher experience, teacher evaluation, and teacher gender in the relationship between students’ BMI status and peer relationships, aiming to provide a more comprehensive understanding of how teacher-related factors shape students’ social interactions.

**Methods:**

Data were drawn from the 2023–2024 academic year of the China Education Panel Survey, comprising 2,002 Chinese middle school students and their homeroom teachers from 100 schools. A two-level HLM model in which Level 1 included students’ BMI status and peer relationships, and Level 2 included teacher experience, teacher evaluation, and teacher gender as cross-level moderating variables.

**Results:**

Students with a normal BMI status were more likely to be accepted by their peers (*β* = 0.836, *p* < 0.001). Teacher experience had a significant positive moderating effect on the association between normal BMI status and peer relationships (*β* = 0.047, *p* < 0.001). Teacher evaluation further amplified this association (*β* = 0.217, *p* < 0.01); that is, the more experienced and the more highly evaluated the homeroom teacher, the stronger the positive impact of a normal BMI status on students’ peer relationships. Teacher gender also significantly moderated this relationship (*β* = 0.246, *p* < 0.05), with the beneficial effect of a normal BMI status on peer relationships being more pronounced in classes taught by female teachers.

**Conclusion:**

Normal BMI status has a significant positive effect on peer relationships, and this association is cross-level moderated by teacher experience, teacher evaluation, and teacher gender. Higher teacher experience, higher teacher evaluation, and classrooms led by female teachers strengthen the positive effect of normal BMI status. These findings highlight the critical role of teacher-related factors in shaping students’ social ecology and provide empirical support for optimizing educational environments and promoting social equity among students.

## 1 Background

In recent years, peer relationships among students have increasingly become a central topic in educational research, particularly regarding the factors that influence students’ social interactions and psychological development. Researchers have commonly focused on the interaction between individual characteristics and social environments (Rubin et al., 2006; Wentzel, 1998). Middle school students, who are in early adolescence, are undergoing a critical developmental stage in which social belonging and peer relationships play an essential role in their mental health and social adaptation (Rubin et al., 2011). Existing studies have shown that Body Mass Index (BMI) status may be an important factor affecting peer relationships: students with a non-normal BMI status (overweight or obese) may experience greater social pressure due to appearance-related concerns, whereas those with a normal BMI status are more likely to be accepted by their peers (Juvonen et al., 2017). However, students’ peer interactions are not determined solely by individual traits; higher-level factors also play an important role. For example, teacher support and a positive classroom climate are typically associated with lower levels of peer bullying, whereas weight-related stigma exposes students with a non-normal BMI status to greater peer pressure. Therefore, teacher-level contextual factors may shape the peer experiences of students with different BMI statuses (Thornberg et al., 2024; Juvonen et al., 2017; Puhl and Latner, 2007).

### 1.1 Student level: impact of student BMI on peer relationships

Peer relationships refer to the degree to which a student is accepted or perceived as popular within the peer group (Roorda et al., 2011). At the student level, BMI status is one of the key factors influencing peer relationships. As an indicator reflecting the ratio of weight to height, BMI status not only represents an individual’s health condition but also significantly shapes others’ first impressions in social interaction contexts. During adolescence, physical appearance and body shape play an especially important role in social encounters, making the influence of student BMI status on peer relationships particularly salient (Berge et al., 2015).

Specifically, students whose BMI status falls within the “normal” or “standard” range are typically perceived as having a healthy body shape that more closely aligns with mainstream appearance norms, and thus are more likely to gain peer recognition and acceptance. This “standard body type” is often associated with positive personality traits such as confidence, proactiveness, and sociability, enabling these students to interact more comfortably and confidently and to obtain greater access to social resources and opportunities (Puhl and Latner, 2007). In contrast, students with a non-normal BMI status (overweight or obese) are more likely to be negatively labeled because their body shape does not fit conventional standards. They may be perceived as “unhealthy,” “lazy,” or “weak yet overweight,” which makes them more vulnerable to ridicule and exclusion and more likely to experience difficulties integrating into mainstream peer groups (Neumark-Sztainer et al., 2002). Overall, the closer a student’s BMI status is to the normal range, the more their appearance and health impression conform to mainstream aesthetic and social expectations, and the more likely they are to be favored and accepted by peers and to develop stronger peer relationships.

### 1.2 Teacher level: interaction between teacher experience and student BMI

Teacher experience refers to the number of years a teacher has served as a classroom instructor (Burroughs et al., 2019). A large body of research has examined the impact of teacher experience on students’ physical and mental development as well as academic performance (Klassen and Chiu, 2010). Teachers with rich teaching experience typically demonstrate higher instructional effectiveness, stronger classroom management skills, and a greater ability to address individual differences among students (Tschannen-Moran and Hoy, 2007). As research has progressed, scholars have increasingly begun to focus on the interaction between teacher experience and student characteristics (such as student BMI status), particularly in the context of students’ social adaptation.

Existing studies indicate that students’ BMI status is closely related to their social adaptation and peer relationships. Students with an abnormal BMI status are more likely to encounter social exclusion or negative evaluations due to body-shape differences, which can undermine their social status and peer relationships within the classroom (Puhl and Latner, 2007; Mylroie, 2013). This impact may place students with an abnormal BMI status at a disadvantage in social situations.

However, more experienced teachers are often able to alleviate the adverse effects of BMI status on students’ social adaptation through inclusive teaching practices and supportive strategies (Dee, 2005). Related research suggests that teachers with extensive teaching experience are more inclined to attend to individual differences among students—including overweight or obese BMI status—by implementing positive classroom management, providing emotional support, and treating students fairly, thereby reducing social difficulties arising from body-shape differences (Jennings and Greenberg, 2009). For example, one study found that teachers with longer teaching experience can mitigate the social challenges faced by students with an abnormal BMI status by strengthening teacher–student interactions and fostering a more inclusive classroom climate, thus exerting a “buffering effect” (Jennings et al., 2013). Taken together, the richer the teacher’s experience, the more it can enhance the positive role of BMI status in students’ social adaptation and peer relationships.

### 1.3 Teacher level: interaction between teacher evaluation and student BMI

Teacher evaluation is an important indicator of the quality of teacher–student interactions and the overall classroom climate, and it can significantly influence students’ academic performance and social development (Roorda et al., 2011). Prior research suggests that in classrooms with higher teacher evaluation, teacher–student interactions tend to be more frequent and positive, which facilitates students’ formation of constructive social ties and, in turn, shapes their popularity within peer relationships (Shek et al., 2021). Moreover, as key shapers of classroom culture, highly regarded teachers not only foster a supportive atmosphere but may also play a central role in guiding students’ social norms. For example, in classrooms where teacher evaluation is high, students are more likely to follow the teacher’s social values, whereas in classrooms with lower teacher evaluation, students’ social behaviors may be more influenced by personal biases or stereotypes (Ryan and Patrick, 2001). Therefore, teacher evaluation may function as an important cross-level moderator between students’ BMI status and peer relationships—specifically, in classrooms with higher teacher evaluation, the positive influence of normal BMI status on students’ social relationships may be more pronounced.

Studies further indicate that classrooms with higher teacher evaluation often show stronger social cohesion and emotional support, which may amplify the effect of students’ normal BMI status on peer relationships, forming a “magnification effect” (Shao et al., 2025). In such classrooms, teachers usually pay closer attention to the quality of peer interactions and actively cultivate a stable and positive social climate, making it easier for students with a normal BMI status to gain peer acceptance. In contrast, in classrooms with lower teacher evaluation, teachers’ influence on students’ social behavior is weaker, and peer interaction patterns may be more shaped by appearance-related stereotypes, thereby weakening the positive impact of BMI status on students’ social relationships (Anderson et al., 2019).

### 1.4 Teacher level: interaction between teacher gender and student BMI

Teacher gender not only shapes teaching styles but may also play an important role in students’ social interactions and classroom management (Sadker and Zittleman, 2009). Male and female teachers can differ in classroom management approaches, patterns of teacher–student interaction, and the level of attention they devote to students’ social development. This is particularly salient in middle school, when students are undergoing rapid socialization and factors such as appearance and body shape become more influential in peer relationships (Puhl and Luedicke, 2012). Given the close link between students’ BMI status and peer relationships, teacher gender may affect the strength of BMI status effects on students’ social dynamics.

Overall, female teachers tend to be more sensitive to students’ social development and emotional needs, and may pay greater attention to students’ appearance, body shape, and their social implications (Roorda et al., 2011). Compared with male teachers, female teachers often emphasize building emotional support systems in the classroom, encourage affective communication among students, and are more likely to foster social interactions that align with mainstream aesthetic norms (Juvonen et al., 2017). In such contexts, students with a normal BMI status may be more likely to gain social advantages. Therefore, the positive effect of normal BMI status on peer relationships may be more pronounced in classrooms led by female teachers.

Although research on gender differences in classroom management remains limited, existing evidence indicates that male teachers tend to be more “controlling” than female teachers (Martin et al., 1998). In these classroom environments, students’ social interactions may be shaped more by individual characteristics such as academic ability or athletic performance, while the role of BMI status is relatively weaker. Accordingly, compared with classrooms led by female teachers, the impact of students’ BMI status on peer relationships may be less evident in classrooms managed by male teachers.

### 1.5 Limitations of previous research and advantages of the present study

In recent years, research in educational psychology has increasingly adopted multilevel (hierarchical) models and multilevel structural equation modeling to address the nested structure of teacher–student data (Thornberg et al., 2024). Building on this methodological trend, the present study centers on an important question that has not yet been systematically tested: whether teacher experience, teacher evaluation, and teacher gender cross-level moderate the association between students’ BMI status and peer relationships.

Second, prior studies have often handled teacher–student matching (ratio) inadequately, which may bias conclusions. Traditional approaches frequently overlook the “one-to-many” nesting inherent in school settings—one teacher typically teaches dozens of students, and class size varies substantially across classrooms. To accommodate single-level analyses (e.g., some SPSS-based regressions), some studies artificially replicate each teacher’s record according to the number of students, forcing a true 1:many structure into 1:1 paired data. This practice changes data independence and weighting, inflates statistical error, and can under- or over-estimate teacher effects. In contrast, hierarchical linear modeling (HLM) accurately represents the “one teacher–many students” hierarchy without altering the original sample ratios. Moreover, HLM assigns teacher-level variables (teacher experience, teacher evaluation, teacher gender) and student-level variables (BMI status, peer relationships) to their appropriate levels, enabling a more precise identification of how teacher-contextual factors may moderate the BMI status–peer relationship through pathways such as teaching style, classroom management, and teacher–student interaction. Accordingly, HLM is employed in this study.

### 1.6 Research objectives and hypotheses

**Figure 1:**
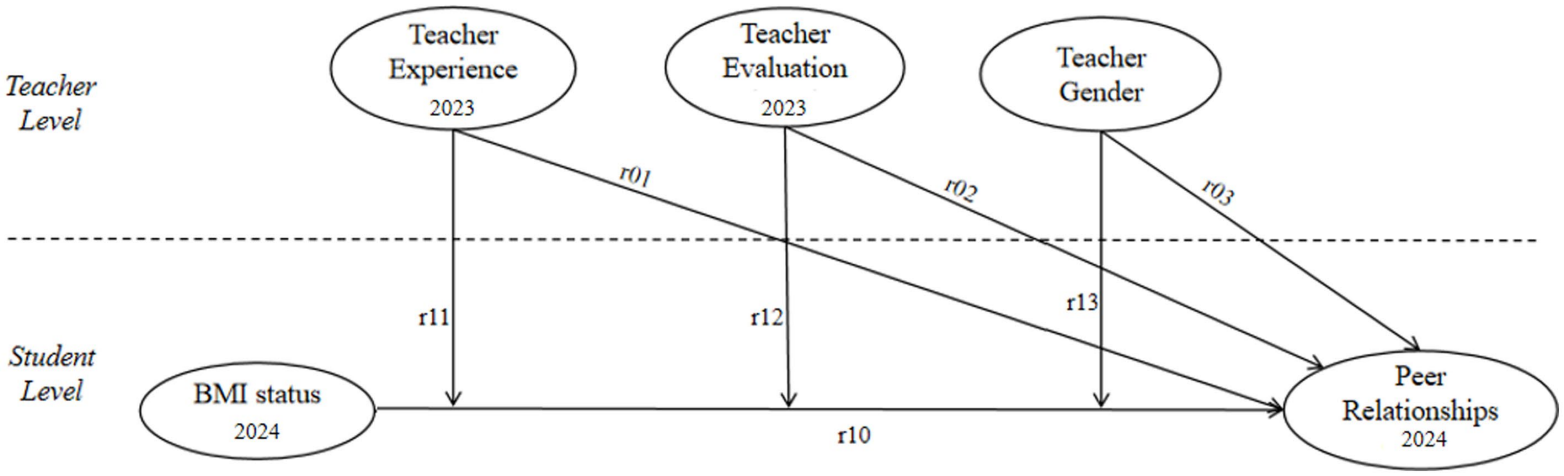
HLM model.

Based on longitudinal HLM data, this study aims to examine how teacher experience, teacher evaluation, and teacher gender serve as cross-level moderators in the relationship between student BMI status and peer relationships, in order to gain a more comprehensive understanding of the mechanisms through which these variables influence students’ social dynamics. Accordingly, the research model (Figure 1) and hypotheses are proposed.

#### Student level (Level-1)

*H1*: Student BMI status has a significant effect on peer relationships; specifically, students with a normal BMI status are expected to show higher peer acceptance/popularity than those who are overweight or obese.

#### Teacher level (Level-2)

*H2*: Teacher experience exerts a significant positive cross-level moderating effect on the relationship between students’ BMI status and peer relationships. Specifically, the greater the teacher’s experience, the more pronounced the peer-relationship advantage of students with a normal BMI status over those who are overweight/obese.

*H3*: Teacher evaluation exerts a significant positive cross-level moderating effect on the relationship between students’ BMI status and peer relationships. Specifically, in classrooms with higher teacher evaluation, students with a normal BMI status are expected to display higher peer acceptance/popularity than their overweight/obese peers.

*H4*: Teacher gender plays a significant cross-level moderating role in the relationship between students’ BMI status and peer relationships. Specifically, compared with male teachers, the peer-relationship advantage of students with a normal BMI status over overweight/obese students is more pronounced in classrooms led by female teachers.

## 2 Methods

### 2.1 Data source

The data used in this study were derived from the China Education Panel Survey (CEPS). This nationally representative and large-scale longitudinal survey was designed and implemented by the China Survey and Data Center at Renmin University of China. Its primary aim is to investigate how family, school, community, and broader social structures influence individual educational outcomes, as well as to explore the mechanisms through which these outcomes shape personal development.

CEPS survey uses the 2023–2024 academic year as its baseline, covering students from Grade 7 to Grade 9 (equivalent to the first to third year of Chinese middle school), across 112 schools and 438 classes. Given the relatively low frequency of educational policy changes and data updates in China, the CEPS dataset is currently one of the most recent and large-scale nationally representative education datasets available. Moreover, national-level surveys typically require substantial resource investment and are difficult to access. Therefore, the comprehensiveness and representativeness of the CEPS data make it a suitable choice for this study, allowing for an effective analysis of the relationships between educational outcomes and related variables.

During data processing, 12 of the initial 112 schools were excluded due to missing data; listwise deletion yielded a final analytic sample of 100 schools. Using class IDs, we treated the 2023 homeroom teacher as the baseline classroom context for the same class in 2024. From each school, one homeroom teacher was randomly selected, and approximately 20 students were randomly drawn from that teacher’s class. The final sample comprised 100 homeroom teachers and 2,002 students. The detailed sample distribution is presented in Table 1.

**Table 1:** Basic information.

Gender	Number/*n* (%)	Weight/kg (SD)	Height/m (SD)	Age/years (SD)		Number/*n* (%)	Age/years (SD)	Experience/year (SD)
Male students	1,015 (50.7%)	78.68 (7.56)	1.68 (0.05)	13.47 (1.13)	Male teachers	39 (39%)	39.10 (7.14)	15.09 (7.15)
Female students	987 (49.3%)	50.80 (4.37)	1.60 (0.03)	13.47 (1.12)	Female teachers	61 (61%)	37.79 (6.25)	13.80 (6.24)

Weekly homeroom-teacher contact hours are unavailable in CEPS; contact time is unobserved.

### 2.2 Variable selection and description

This study adopts teacher experience, teacher evaluation, and teacher gender from 2023, together with students’ BMI status and peer relationships from 2024, as the core variables based on clear logical reasoning. First, teacher experience measured in 2023 reflects teachers’ potential long-term influence on students. Although teaching experience is relatively stable over time, it plays a meaningful role in shaping students’ peer relationships and may affect the cumulative social behaviors that students exhibit in 2024. Thus, a key aim of this study is to examine how “teachers’ prior characteristics” may exert potential effects on students’ subsequent social functioning. Second, teacher evaluation in 2023 (i.e., students’ ratings of teacher popularity) serves as an important indicator of the quality of teacher–student relationships. It captures the interactional climate between teachers and students and implies that teachers’ social standing and classroom influence may carry over to shape students’ peer relationships in the following year. With respect to students’ BMI status in 2024, students’ current physical condition is especially salient in school contexts where appearance or body shape may directly affect peer interactions. Although teacher experience may indirectly shape students’ health behaviors and self-perceptions—and thereby influence BMI status in 2024—using the most recent BMI status data allows us to capture the immediate effect of body shape on peer interactions within the same year. Therefore, selecting peer relationships in 2024 as the dependent variable is reasonable and consistent with causal-inference logic, ensuring temporal alignment between predictors and outcomes.

Specifically, at the student level, the independent variable is students’ BMI status (BMI = weight in kilograms/height in meters^2^). Because there were no underweight students (BMI ≤ 18.5) in this sample, the underweight category was excluded, and BMI status was then dichotomized following prior studies (Puhl and Latner, 2007; Juvonen et al., 2017): 0 = overweight/obese status (BMI ≥ 25; n = 1,027; 51.3%) and 1 = normal status (18.5 < BMI < 25, n = 975; 48.7%). The dependent variable is peer relationships, assessed with a single item on a 1–10 scale (1 = lowest, 10 = highest) indicating the student’s standing among peers. At the teacher level, the cross-level moderators include teacher experience (years of teaching; range 1–25, with higher values indicating greater experience), teacher evaluation (teacher popularity within the school; single item, 1–7), and teacher gender (0 = male homeroom teacher, 1 = female homeroom teacher). Teacher-level and student-level data were collected via the corresponding teacher and student questionnaires, respectively.

### 2.3 Model specification and description

Based on the HLM model structure in Table 2 and the corresponding parameter interpretations, we organized the relationships among HLM variables and coefficients to more intuitively illustrate the effects of each variable on students’ peer relationships.

**Table 2:** HLM variables and equations.

Model	Level	Equations
Unconditional model	Student level	Peer relationships *_ij_ = β_0j_ + r_ij_*
	Teacher level	*β_0j_ = γ_00_ + U_0j_*
Random-coefficient model	Student level	Peer relationships *_ij_ = β_0j_ + β_1j_(BMI* status*)_ij_ + r_ij_*
	Teacher level	*β_0j_ = γ_00_ + U_0j_, β_1j_ = γ_10_ + U_1j_*
Conditional/full model	Student level	Peer relationships *_ij_ = β_0j_ + β_1j_(BMI* status*)_ij_ + r_ij_*
	Teacher level	*β_0j_ = γ_00_ + γ_01_* teacher experience *_j_ + γ_02_* teacher evaluation *_j_ + γ_03_* teacher gender *_j_ + U_0j_* *β_1j_ = γ_10_ + γ_11_* ***teacher experience*** *_j_ + γ_12_* ***teacher evaluation*** *_j_ + γ_13_* ***teacher gender*** *_j_ + U_1j_*

*Bold italic*, grand-mean centering.

Unconditional model is primarily used to analyze students’ peer relationships at the individual level, without considering BMI status or other factors, thereby reflecting the basic structure of peer relationships. The random-coefficient model further examines the effect of student BMI status on peer relationships and allows this effect to vary across schools, capturing its random effect. The conditional model incorporates teacher-level variables (teacher experience, teacher evaluation, and teacher gender) to investigate how these factors influence the model intercept (*β₀_j_*) and the effect of BMI status on peer relationships (*β₁_j_*). This model emphasizes the role of teacher-level factors in moderating the impact of BMI status. Through Table 2, we can intuitively understand how teacher-level factors, student BMI status, and their interactions jointly influence students’ peer relationships.

### 2.4 Data analysis

To examine the basic characteristics of the data, SPSS 23.0 was used to conduct descriptive statistics, and multicollinearity was assessed through correlation analysis. To present more intuitively the combined effects of variables on students’ peer relationships within the HLM framework, hierarchical linear modeling was performed using HLM 7.0 (Raudenbush et al., 2011). Before the formal analysis, BMI indices were first calculated and processed in Excel; all variables were then classified into student-level and teacher-level factors, and the BMI index was dichotomized into overweight/obese status versus normal status prior to estimating the corresponding regression coefficients. All statistical analyses were completed with SPSS 23.0 and HLM 7.0, and the significance level was set at 0.05.

## 3 Results

### 3.1 Correlation and descriptive statistics

The correlation analysis results for the variables included in the model are presented in Table 3. VIF for teacher experience = 1.28, for teacher evaluation = 1.27, and for teacher gender = 1.01; since there is only one Level-1 predictor (BMI status), its VIF = 1.00. All values are well below conventional cutoffs (e.g., 5 or 10), indicating no multicollinearity concerns. Generally, when the absolute value of the correlation coefficient between variables exceeds 0.70, high correlation may be present (Field, 2024). In this study, the absolute values of correlation coefficients ranged from 0.001 to 0.716. Only one pair of variables showed a correlation slightly above 0.70, but still within an acceptable range. Therefore, the issue of multicollinearity is considered to be minimal (Byrne, 2013).

**Table 3:** Correlation and descriptive statistics.

Level	Variable	Student (*n* = 2002)		Teacher (*n* = 100)		
		1	2	3	4	5
Student level	1. BMI status	1				
	2. Peer relationships	0.361**	1			
Teacher level	3. Teacher experience	0.001	0.500**	1		
	4. Teacher evaluation	0.141**	0.716**	0.459**	1	
	5. Teacher gender	−0.021	0.034	−0.088**	0.014	1
*M*		0.49	5.31	14.26	4.48	0.61
*SD*		0.5	1.228	6.555	1.038	0.488
Skewness		0.052	0.257	−0.139	0.068	−0.449
Kurtosis		−1.999	−0.204	−1.035	−0.038	−1.8

** *p* < 0.01.

The mean values (*M*) of the five variables ranged from 0.49 to 14.26, indicating that all variables—from teacher experience to students’ peer relationships—fell within the positive evaluation range. The absolute values of skewness ranged from 0.052 to 0.449, all below 2; the absolute values of kurtosis ranged from 0.038 to 1.999, all below 8.00. Therefore, according to established criteria in prior studies, the data can be regarded as approximately normally distributed (Kline, 2023; Miao and Zhang, 2024).

As shown in Table 3, the correlation coefficient between student BMI status and peer relationships is 0.361 (*p* < 0.01), indicating a significant positive correlation. The correlation between student BMI status and teacher evaluation is 0.141 (*p* < 0.01), also showing a significant positive relationship. At the teacher level, the correlation coefficient between teacher experience and peer relationships is 0.5 (*p* < 0.01), demonstrating a significant positive correlation. The correlation between teacher evaluation and peer relationships is 0.716 (*p* < 0.01), indicating a strong positive association. These results suggest that teacher-level variables such as teacher experience and teacher evaluation may be related to student-level peer relationships, implying potential cross-level effects. However, since correlation analysis only reveals linear relationships between variables, specific cross-level effects need to be further tested using HLM.

### 3.2 Model fit comparison

As shown in Table 4, we evaluated model improvement along the planned nesting sequence. Moving from the Unconditional (intercept-only) model to the Random-coefficient model (with a random BMI status slope) reduced deviance from 5113.75 to 4494.61, yielding *Δχ^2^*(2) = 619.14, *p* < 0.001, which indicates meaningful between-class variability in the BMI status effect and justifies estimating a random slope. Adding Level-2 moderators to obtain the Conditional/full model further reduced deviance from 4494.61 to 4092.48, *Δχ^2^*(2) = 402.13, *p* < 0.001, showing that teacher-level predictors provide additional explanatory power beyond random slopes. Overall, the jump from Unconditional to Conditional/full (5113.75 → 4092.48) produced *Δχ^2^*(4) = 1021.27, *p* < 0.001, confirming a substantial and statistically significant improvement in fit. These results indicate that introducing Teacher Experience, Teacher Evaluation, and Teacher Gender materially enhances the model’s ability to account for students’ peer relationships.

**Table 4:** Model fit comparison.

Model comparison	Deviance value	Difference		*χ* ^2^
		*df*	Deviance	
Unconditional → random-coefficient	5113.75 → 4494.61	2	619.14	*p* < 0.001
Random-coefficient → conditional/full	4494.61 → 4092.48	2	402.13	*p* < 0.001
Unconditional → conditional/full	5113.75 → 4092.48	4	1021.27	*p* < 0.001

### 3.3 Hierarchical effect analysis

Table 5 presents the results of the HLM analysis. The intra-class correlation coefficients (*ICC*) from the unconditional model indicate that *ICC(1)* = 0.580 > 0.059 and *ICC(2)* = 0.965 > 0.7, suggesting that the data are suitable for further HLM analysis (Castro, 2002).

**Table 5:** HLM analysis results.

Effect	Predictor	Parameter (γ)	Peer relationships		
			*β*	*SE*	*t*
Fixed effect					
*Intercept* *β_0_*		*γ_00_*	4.895***	0.035	141.804
	Teacher experience	*γ_01_*	0.053***	0.006	8.366
	Teacher evaluation	*γ_02_*	0.785***	0.044	17.472
	Teacher gender	*γ_03_*	0.001	0.065	0.016
*Slope* *β_1_*	BMI status	*γ_10_*	0.836***	0.050	16.580
	BMI status × teacher experience	*γ_11_*	0.047***	0.008	−5.305
	BMI status × teacher evaluation	*γ_12_*	0.217**	0.068	3.189
	BMI status × teacher gender	*γ_13_*	0.246*	0.097	2.521
Random effect					
	LEVEL 1	*r*	0.393		
	LEVEL 2	*Intercept, u_0_*	0.083***		
	LEVEL 2	BMI *slope, u_1_*	0.182***		
Unconditional model *ICC(1)*			0.580		
Unconditional model *ICC(2)*			0.965		
*F^2^*_within-group_			0.385(38.5%)		
*F^2^*_between-group_			0.906(90.6%)		

* *p* < 0.05, ** *p* < 0.01, *** *p* < 0.001; ICC are computed from the unconditional (null/random-intercept) model using variance components only. The formula for ICC(1) in the unconditional model is as follows: $\mathrm{ICC}(1)=\tau_{0}/(\tau_{0}+\sigma^{2})$ .

Variance-reduction index $F^{2}$ compares the full model to the baseline/null: $F_{\text{within}/\text{between}}^{2}=(\tau^{2}/\sigma_{\text{baseline}}^{2}-\tau^{2}/\sigma_{\text{full}}^{2})/\tau^{2}/\sigma_{\text{baseline}}^{2}$ .

According to Cohen (2013) criteria, an $F^{2}$ value greater than 0.35 indicates a strong effect. The within-group improvement ratio was 38.5% ( $F^{2}$ = 0.385), and the between-group improvement ratio was 90.6% ( $F^{2}$ = 0.906), both of which are significantly greater than 35%. This suggests that the comprehensive model shows a substantial improvement over the baseline model in explaining the data, with very high explanatory power.

**Figure 2:**
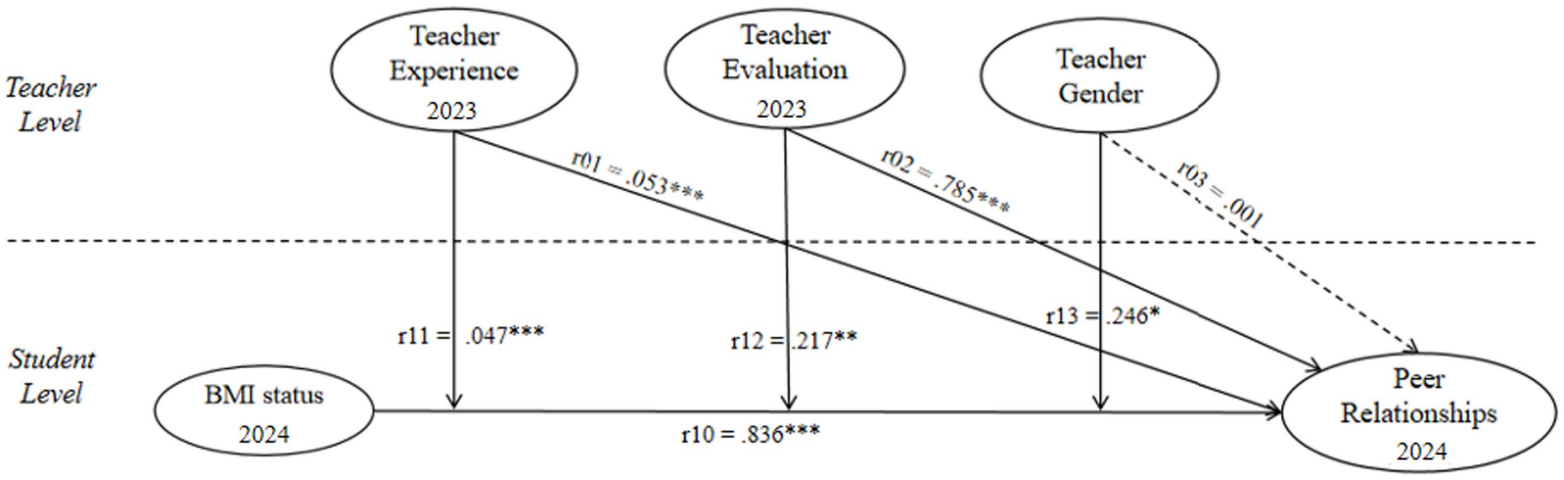
HLM model path diagram.

As shown in Table 5 and Figure 2, the fixed effects from the HLM analysis indicate that the intercept (*γ_00_* = 4.895, *p* < 0.001) represents the average level of students’ peer relationships when all predictors are at their mean values. Teacher experience (*γ_01_* = 0.053, *p* < 0.001) has a significant positive effect on peer relationships, suggesting that students tend to report better peer relationships when their homeroom teachers have more teaching experience. Teacher evaluation (*γ_02_* = 0.785, *p* < 0.001) also shows a significant positive effect, indicating that higher student evaluations of the teacher are associated with more positive peer relationships in the classroom. The main effect of student BMI status (*γ_10_* = 0.836, *p* < 0.001) demonstrates that, holding other variables at their average levels, students with a normal BMI status generally exhibit better peer relationships, thereby supporting *H1*.

**Figure 3:**
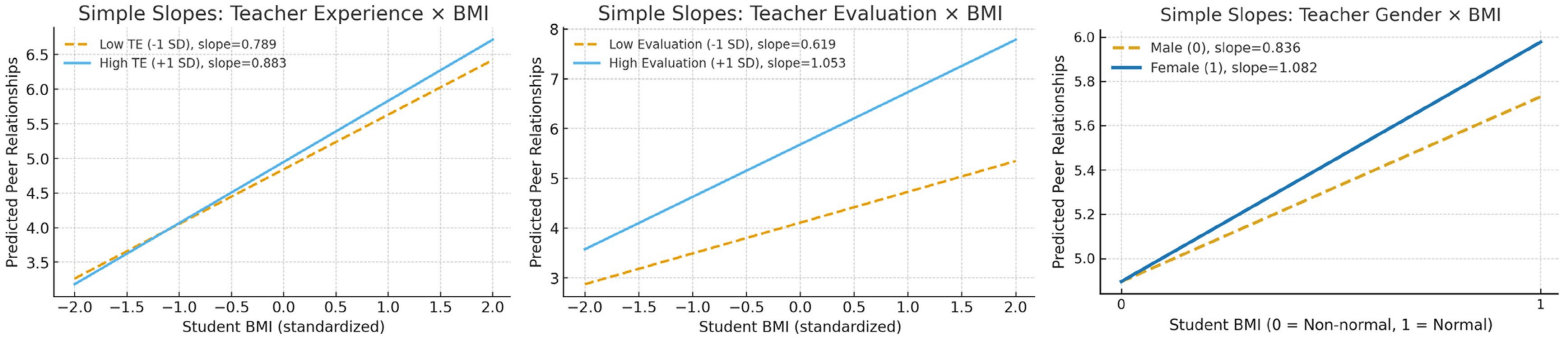
Simple slopes plot.

Regarding the interaction effects (see Table 5; Figure 3), the cross-level interaction between student BMI status and teacher experience (*γ_11_* = 0.047, *p* < 0.001) is statistically significant. This indicates that as teacher experience increases, the positive effect of a normal BMI status on students’ peer relationships becomes stronger, whereas the negative effect associated with a non-normal BMI status becomes weaker, supporting *H2*. The interaction between teacher evaluation and student BMI status (*γ_12_* = 0.217, *p* < 0.01) is also significant, showing that in classrooms with higher teacher evaluation, the effect of students’ normal BMI status on peer relationships is more pronounced, thereby confirming *H3*. In addition, the interaction between student BMI status and teacher gender (*γ_13_* = 0.246, *p* < 0.05) reaches significance, suggesting that female teachers play a more salient moderating role in the association between students’ normal BMI status and peer relationships, supporting *H4*.

The random-effects results in Table 5 show that the residual variance at the student level is 0.393, indicating that some unexplained variation remains at Level 1. At the teacher level, the random effect of the intercept (*u_0_* = 0.083, *p* < 0.001) is significant, suggesting that teachers differ in their overall influence on students’ peer relationships. The random effect of the BMI status slope (*u_1_* = 0.182, *p* < 0.001) is likewise significant, indicating cross-teacher variability in how BMI status relates to peer relationships.

Overall, both the fixed and random effects demonstrate that student BMI status, teacher experience, teacher evaluation, and teacher gender significantly shape students’ peer relationships, and that these effects vary across teachers. The model shows good overall fit and effectively explains variance at both the student and teacher levels.

## 4 Discussion

This study employed HLM to examine the moderating roles of teacher experience, teacher evaluation, and teacher gender in the association between students’ BMI status and peer relationships. The results indicated that normal BMI status was significantly and positively related to better peer relationships, suggesting that students within the normal BMI range are more likely to gain peer acceptance. In addition, teacher experience and teacher evaluation showed significant positive effects on students’ peer relationships; notably, classrooms in which teachers were more highly evaluated by students tended to exhibit a more positive peer-interaction climate. However, the main effect of teacher gender was not statistically significant, implying no direct difference between male and female teachers in their impact on students’ peer relationships.

At the teacher level, we identified robust cross-level moderating effects: the positive association between normal BMI status and peer relationships was significantly strengthened as teacher experience increased, as teacher evaluation rose, and in classrooms taught by female teachers. These findings suggest that teacher characteristics not only exert direct influences on students’ social outcomes but also indirectly shape the peer environment by amplifying the impact of BMI status on peer relationships.

### 4.1 Effect of student BMI on peer relationships

The results of this study support *H1*, indicating that students’ normal BMI status has a significant impact on peer relationships. Specifically, students with a normal BMI status tend to report more positive and favorable peer relationships. This finding aligns with prior research emphasizing the importance of appearance and body shape in adolescent social interactions (Strauss and Pollack, 2003; Juvonen et al., 2017). Particularly during the middle school years, peer groups gradually become the primary context for students’ socialization, and physical appearance plays a more salient role in interpersonal relationships (Cillessen and Marks, 2011). One possible explanation is that adolescents are more likely to make social choices based on observable external characteristics such as appearance and body type, which may produce clear patterns of social preference or exclusion.

Students with an overweight/obese BMI status are often more likely to be labeled with negative stereotypes such as “lazy” or “lacking self-control,” increasing their risk of peer rejection (Neumark-Sztainer et al., 2002; Puhl and Latner, 2007; Giel et al., 2010). In addition, overweight or obese students may experience greater difficulties participating in physical activities or group games, which can reduce their opportunities to integrate into peer groups (Strauss and Pollack, 2003). In contrast, students with a normal BMI status are more likely to receive positive evaluations and acceptance from peers because their appearance aligns more closely with prevailing social aesthetic standards, giving them a relative social advantage in peer interactions (Juvonen et al., 2017). Therefore, BMI status emerges as a critical factor shaping the quality of peer relationships, which is highly consistent with the conclusions of this study.

### 4.2 Interaction between student BMI and teacher experience

The findings support *H2*, indicating that teacher experience significantly moderates the relationship between students’ BMI status and peer relationships. Specifically, as teacher experience increases, the positive association between normal BMI status and peer relationships becomes progressively stronger. This suggests that more experienced teachers can more effectively shape BMI-related social interaction processes, thereby reinforcing the beneficial impact of a normal BMI status on students’ peer relationships.

On the one hand, this pattern may be explained by the fact that veteran teachers have accumulated more effective strategies for classroom management and teacher–student interaction (Klassen and Chiu, 2010; Tschannen-Moran and Hoy, 2007). They are more sensitive to appearance- or body-shape-based negative judgments and social exclusion, and can intervene in a timely manner to foster a more positive and inclusive peer environment (Dee, 2005). On the other hand, experienced teachers often hold more comprehensive educational philosophies and are more inclined to cultivate students’ acceptance and understanding of others, which helps reduce bias and discriminatory behaviors toward students with a non-normal (overweight/obese) BMI status, thereby easing the social pressure these students face (Morin et al., 2011; Moy, 2015).

This study also suggests that teacher experience is not only a factor promoting academic performance but also plays an important cross-level role in students’ social adaptation and peer development. When teachers are more experienced, classrooms tend to exhibit a more inclusive and positive climate; such teachers are more likely to build a fair class culture that respects individual differences, enabling students to develop peer relationships grounded in mutual acceptance and understanding (Wang et al., 2022).

### 4.3 Interaction between student BMI and teacher evaluation

The results of this study verified *H3*, indicating that teacher evaluation significantly moderates the relationship between students’ BMI status and peer relationships. Specifically, in classrooms where teachers receive higher evaluations from students, the positive effect of a normal BMI status on peer relationships is significantly stronger. This finding further underscores the pivotal role of positive teacher–student interactions in shaping students’ social ecology.

Teacher evaluation essentially reflects the quality of teacher–student interactions; higher ratings indicate more harmonious relationships and greater student recognition and respect for the teacher (Hughes, 2011). In such classroom contexts, teachers are more likely to cultivate a positive emotional climate and encourage mutual acceptance and respect among students. Consequently, students tend to be more accepting of peers who conform to mainstream aesthetic standards, and those with a normal BMI status are more likely to receive favorable peer feedback and enjoy social advantages in these environments (Jones et al., 2004; Hamre and Pianta, 2001).

Moreover, this study further reveals that highly evaluated teachers can amplify the positive influence of normal BMI status on students’ social functioning. This may be because teachers’ role modeling and emotional support strengthen an atmosphere of peer acceptance, thereby reducing the negative impact of appearance-related factors on student interactions (Wentzel, 2002). At the same time, classrooms with high teacher evaluations often show greater cohesion; more frequent teacher–student interactions help foster a more inclusive and positive classroom culture, encouraging students to focus on individual strengths rather than superficial body-shape characteristics (Pianta and Stuhlman, 2004).

### 4.4 Interaction between student BMI and teacher gender

The results of this study partially support *H4*, showing that teacher gender significantly moderates the pathway from students’ BMI status to peer relationships. Specifically, in classrooms taught by female teachers, students with a normal BMI status tend to display a more pronounced peer advantage. This pattern should be understood as reflecting contextual differences in classroom interaction styles and communication orientations rather than any essentialized interpretation of gender; substantial individual variation exists among teachers of all genders.

A neutral socio-cultural explanation is that teachers of different genders may, on average, adopt somewhat different socio-emotional communication styles and empathy orientations. Prior studies suggest that female teachers often show higher sensitivity and responsiveness in emotional interaction and support, which may make appearance-related social cues more likely to enter classroom interactions and peer-evaluation processes, thereby accelerating the formation of peer feedback (Liang et al., 2022). In contrast, in some contexts male teachers may place greater emphasis on more visible indicators such as task demands, discipline, or academic performance, so subtle peer dynamics linked to BMI status or appearance may be less salient at the classroom level. Importantly, these differences represent context-dependent average tendencies rather than fixed or unidirectional gender effects.

Teacher gender may also shape classroom climate, thereby influencing the norms and thresholds that guide peer interactions. In classrooms that emphasize relationship building and socio-emotional support, students who align more closely with mainstream appearance norms (e.g., those with a normal BMI status) may receive more immediate positive peer feedback and gain structural advantages within peer networks. At the same time, such climates may inadvertently increase the risk of social marginalization for students who are overweight or obese. Therefore, educational practice should avoid emphasizing or reinforcing any “appearance standard” and instead adopt equity- and inclusion-oriented classroom management across all classes (Liang et al., 2022).

Finally, caution is warranted when generalizing this moderating effect. The observed pattern may co-vary with subject domain, school norms, teacher experience, or classroom composition, and future studies should further examine these boundary conditions.

## 5 Research contributions and practical implications

This study employed HLM to investigate the cross-level moderating effects of teacher experience, teacher evaluation, and teacher gender on the relationship between students’ BMI status and peer relationships, thereby extending the research perspective on BMI status and social relationships. The results indicate that when teachers have more teaching experience, the positive association between normal BMI status and peer relationships is strengthened, suggesting that experienced classroom management can enhance the beneficial role of normal BMI status in students’ social interactions. When teacher evaluation is higher, the positive effect of normal BMI status on peer relationships is further amplified, implying that teacher popularity may reinforce the influence of BMI-status–related appearance cues in students’ social dynamics. In addition, the effect of normal BMI status on peer relationships is more pronounced in classrooms led by female teachers, indicating that teacher gender may shape students’ sensitivity to BMI status differences. Overall, these findings not only enrich theoretical understanding of how teacher-related factors shape students’ social development but also provide important practical implications for school education and classroom management.

## 6 Research limitations and future directions

Using the 2023–2024 CEPS data, our findings are nationally representative; however, as schooling contexts continue to evolve, their generalizability to current educational settings should be further examined. The present models primarily foreground teacher-level influences while omitting other important determinants (e.g., family environment and socioeconomic background). Therefore, even with HLM and the cross-year design (teacher variables in 2023; student outcomes in 2024), residual confounding and potential bidirectional associations may remain. Future studies are encouraged to use multi-wave longitudinal panels and to apply lagged controls and cross-lagged frameworks (e.g., RI-CLPM) for more rigorous testing. In addition, students’ BMI status and peer relationships were measured in the same wave, which raises concerns about simultaneity and possible reverse pathways. In CEPS, peer relationships are student self-reports and do not include teacher ratings or sociometric nominations; thus, future work should incorporate more objective health indicators and multi-informant peer measures to reduce common-method bias. Several key constructs in this study (e.g., teacher evaluation and peer relationships) were assessed with single items; employing validated multi-item scales would improve measurement quality. BMI status was treated as a 2024 cross-sectional indicator, so year-to-year changes in BMI status could not be captured. Finally, CEPS lacks classroom-level information such as weekly homeroom-teacher contact hours and subject content/health-education implementation. Accordingly, we interpret classroom-level moderation conservatively and recommend that future research add timetable- and subject-level metadata to enable more comprehensive analyses.

## 7 Conclusion

Drawing on CEPS data, this study employed HLM to examine the cross-level moderating effects of teacher experience, teacher evaluation, and teacher gender on the relationship between students’ BMI status and peer relationships. The results show that students with a normal BMI status enjoy clear advantages in peer relationships. Moreover, greater teacher experience strengthens the positive impact of normal BMI status on students’ social interactions; higher teacher evaluation further amplifies the positive association between normal BMI status and peer relationships; and this beneficial effect is more pronounced in classrooms taught by female teachers. These findings not only illuminate the complexity of how BMI status shapes students’ social relationships, but also highlight the critical moderating roles of teacher-related factors. Accordingly, we recommend that schools enhance teacher training, optimize classroom management, and advance social-equity education to cultivate a more inclusive peer environment, reduce social inequalities linked to BMI status differences, and ultimately improve students’ social adaptability and the overall quality of the school climate.

## Data Availability

Publicly available datasets were analyzed in this study. The datasets generated and/or analyzed during the current study are publicly available in Figshare and can be accessed via the following link: https://doi.org/10.6084/m9.figshare.30172891.
